# Data-driven Classification of the 3D Spinal Curve in Adolescent Idiopathic Scoliosis with an Applications in Surgical Outcome Prediction

**DOI:** 10.1038/s41598-018-34261-6

**Published:** 2018-11-02

**Authors:** Saba Pasha, John Flynn

**Affiliations:** 1 Division of Orthopedic Surgery, Children’s Hospital of Philadelphia, Philadelphia, PA 19141 USA; 2 Department of Surgery, University of Pennsylvania, Philadelphia, PA 19141 USA

## Abstract

Adolescent idiopathic scoliosis (AIS) is a three-dimensional (3D) deformity of the spinal column. For progressive deformities in AIS, the spinal fusion surgery aims to correct and stabilize the deformity; however, common surgical planning approaches based on the 2D X-rays and subjective surgical decision-making have been challenged by poor clinical outcomes. As the suboptimal surgical outcomes can significantly impact the cost, risk of revision surgery, and long-term rehabilitation of adolescent patients, objective patient-specific models that predict the outcome of different treatment scenarios are in high demand. 3D classification of the spinal curvature and identifying the key surgical parameters influencing the outcomes are required for such models. Here, we show that K-means clustering of the isotropically scaled 3D spinal curves provides an effective, data-driven method for classification of patients. We further propose, and evaluate in 67 right thoracic AIS patients, that by knowing the patients’ pre-operative and early post-operation clusters and the vertebral levels which were instrumented during the surgery, the two-year outcome cluster can be determined. This framework, once applied to a larger heterogeneous patient dataset, can further isolate the key surgeon-modifiable parameters and eventually lead to a patient-specific predictive model based on a limited number of factors determinable prior to surgery.

## Introduction

Adolescent idiopathic scoliosis (AIS) is a three-dimensional (3D) deformity of the spinal column with an onset around puberty^1–3^. The pathomechanisms associated with the development and progression of the AIS remain unclear^1,3^, yet non-surgical and surgical treatment options have been proved effective to prevent the spinal deformity progression^2^. The most common surgical procedure, posterior spinal fusion, realigns a part or the entire spine and secures it with a metal rod posteriorly, resulting in ossification of the vertebral bodies and stabilization of the curve^4^. The success of the surgical treatment of AIS is evaluated with function and mobility, patient’s postural balance, primary or compensatory curve progression, patients’ satisfaction, and quality of life at long-term follow-ups^5–11^.

In order to guide the surgical planning, including selecting the fusion levels, de-rotation of the spine, and imparting the sagittal curvature of the spine (kyphosis, lordosis), several guidelines based on the shape of the spine before surgery have been developed^12–14^. As the two- dimensional (2D) spinal X-rays remain the mainstay in clinical diagnosis of the AIS patients, the surgical decision making guidelines are mainly based on the 2D shape of the spine, resulting in potentially erroneous assessment of the curve^15,16^. Furthermore, identifying the curve types remain largely subjective, influenced by the observer interpretation, and inter-observer variability^17–20^. As a result, the surgical decision-making and the treatment outcome prediction of the scoliotic curves, particularly in patients who cannot easily be assigned to a specific group through the current classification systems, have been challenged by suboptimal outcomes^21–23^. To this date, an objective method that can describe the 3D outcome of the scoliosis surgery at long-term follow-ups by taking into consideration the pre-operative shape of the spine and the surgical intervention has not been fully developed^9^. The successful prediction of the outcome can improve the patient satisfaction and reduces the costs and risks associated with a need for revision surgery^24^.

As the 3D curvature of the spine can explain a large amount of variability in the spinal alignment among the patients, a 3D classification of the overall spinal curve, as opposed to the segmental uni-planar measurements of the curve, can specify the true curvature of the spinal deformity in AIS patients. Similarly, the 3D surgical outcome evaluation of spine can assure that the harmony of the spinal configuration in the three anatomical planes is not overlooked^9^. Patient classification based on the 3D spinal curve, both pre- and post-operatively, can reduce the errors associated with the *subjective* 2D classification by an *objective* use of the 3D overall shape of the spine.

The current study aims to develop an objective, data-driven framework for classification-based outcome prediction of the spinal surgery in AIS. More specifically, the study herein focuses on determining *pathways*, comprised of the variables that can be identified prior to the surgery or modified during the surgery, and statistically examine the association of these *pathways* to the 3D shape of the spine at two-year follow-ups. The underlying assumption of this approach is that the biomechanical changes induced during the surgery can be described using a combination of the 3D shape of the spine at early post-operative and the location of the vertebral levels that are immobilized via a metal rod and are fused within two-year post-operative thus changing the mechanical behaviour of the spine in the instrumented section.

**Figure 1:**
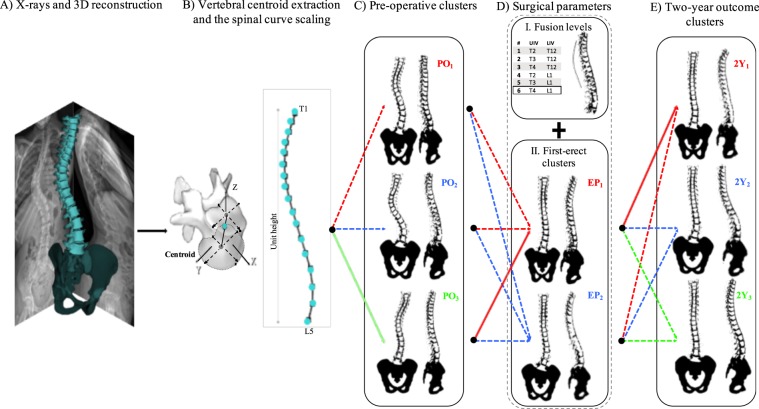
The framework for 3D classification, path assignment, and outcome determination: (**A**) the 3D reconstruction of the spine from bi-planar X-rays is generated. (**B**) The vertebral centroids are calculated. The spline is generated by linearly interpolating the T1 to L5 spinal vertebral centroids 3D positions and is scaled in all directions (i.e., isotropically) such that the unit height is achieved. (**C**) K-means clusters are used to group the pre-operative (PO) 3D scaled spinal curves. Three clusters are identified in our cohort (see Methods). (**D**) I. Fusion levels (F) are specified based on the position of the upper and lower fused vertebrae (UIV and LIV). Six fusion levels are identified in our cohort. II. K-means clusters are used to group the early post-operative (EP) 3D scaled spinal curvatures. Two clusters are identified in our cohort. (**E**) K-means clusters are used to group the 3D scaled spinal curves at two-year follow-up (2Y). Three clusters are identified in our cohort. The assigned *treatment path* for one patient has been shown with solid lines, indicating that PO_3_-EP_1_-F_6_ path leads to 2Y_1_ cluster at two-year follow-up (see Methods). The sagittal, frontal, and axial views of the PO, EP, and 2Y clusters are shown in Fig. 3. The 3D shapes of these clusters are shown in Fig. 4.

To identify these *pathways* and their relationships with the outcome of surgery, we hypothesize that the combination of the 3D curvature of the spine before surgery and the biomechanical impact of the surgery on the spinal alignment (the early post-operation 3D shape of the spine plus the fusion levels) can significantly determine the 3D shape of the spine at two-year follow-ups in the AIS population (Fig. 1).

## Methods

### Patient population

A total number of 67 AIS with a main right thoracic curve (Lenke 1 and 2) age between 10–18 years were selected consecutively and retrospectively. All patients were selected from one hospital center, the Children’s Hospital of Philadelphia, who underwent a posterior selective thoracic fusion with segmental derotation of the vertebrae. All patients had biplanar spinal stereoradiography at three visits: the X-ray images were registered within one-week pre-operation (PO), early (within a month) post-operation (EP), and at two-year after surgery (2Y). The exclusion criteria were previous spinal surgery, vertebral supernumerary, neuromuscular conditions, and musculoskeletal conditions other than scoliosis. Twenty non-scoliotic adolescents verified by bi-planar spinal X-rays and clinical examinations retrospectively were included as the control group. The institutional review board (IRB) at the Children’s Hospital of Philadelphia approved the study procedures and the research was performed in accordance with the relevant guidelines and regulations. A waiver of consent/parental agreement was granted by the IRB.

### Data collection and compilation

The 3D reconstruction of the spine was generated in a commercially available software, SterEOS 2D/3D (EOS imaging, Paris, France) for pre- and post-operative X-rays^25,26^. The 3D reconstruction of the vertebral bodies was used to calculate the 3D coordinates of the vertebral centroids (X, Y, Z) in the global coordinate system of the spine^27^. An isotropic scale factor, in three dimensions, was used to normalize the spinal height [0–1] of each patient in the AIS cohort (Fig. 1). The same process, i.e., 3D reconstruction, center extraction, and scaling was performed for the control cohort. The Z levels of the average scaled spines in controls were used to interpolate the (X, Y) coordinate of the consecutive vertebral centroids in the AIS cohort, resulting in obtaining (X, Y) coordinates at equal Z levels for all the patients. This two-step normalization process was performed to scale all the spines in the Z-direction, eliminating to need to process the variabilities in the Z-direction and make comparison between the curves at the same Z-levels possible.

### Patient classification

A K-means clustering method^28^ was used to cluster the 3D scaled scoliotic spines. Given that the Z coordinates of the T1-L5 spinal vertebrae for all patients are the same (as described above) the clustering is performed only on (X, Y) coordinates of the vertebrae of all patients. The number of clusters was determined by calculating the silhouette values using the Euclidian distances^29^. The K-means cluster analysis was performed on the scaled PO, EP, and 2Y spines resulting in determining clusters of patients at the three time-points.

The fusion levels i.e., the upper and lower instrumented vertebrae (UIV and LIV) were recorded. All fusion levels were determined and a group number was assigned to each distinct fusion level, i.e., same UIV *and* LIV. The *treatment paths* were then determined using the PO cluster number (PO_i_), EP cluster number (EP_j_), and fusion level group (F_m_), constructing a three-level *path* presented as PO_i_-EP_j_-F_m_ (Fig. 1).

A multinomial regression^30^ model was used to predict the outcome cluster at two-year (2Y_k_) from the treatment paths for the cohort as follows:

$$f({{\rm{PO}}}_{{\rm{i}}},{{\rm{EP}}}_{{\rm{j}}},\,{{\rm{F}}}_{{\rm{m}}})\to \,2{{\rm{Y}}}_{{\rm{k}}}$$

where i, j, k are the cluster number at each given time-point and m is the fusion group. The number of patients in each *treatment path* that had the same 2Y_k_ was calculated to determine the occurrence of certain outcome for each of the identified *treatment paths*.

**Figure 2:**
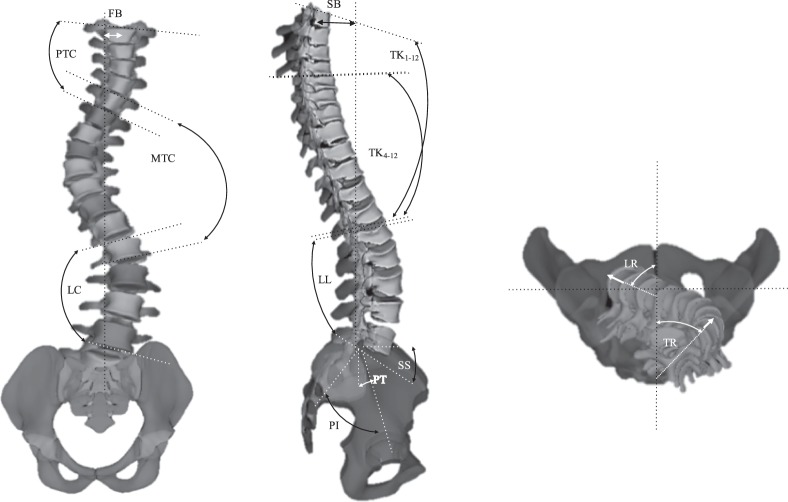
Visual presentation of the radiographic measurements of the spinal deformities that are being used for clinical evaluation of the patients. (**A**) In *frontal* view: proximal thoracic Cobb (PTC), main thoracic Cobb (MTC), lumbar Cobb (LC), and frontal balance (FB). (**B**) In *sagittal* view: T1-T4 and T1-T12 thoracic kyphosis (TK), lumbar lordosis (LL), pelvic incidence (PI), sacral slope (SS), pelvic tilt (PT), and sagittal balance (SB). (**C**) The 3D model of the spine is used for axial measurements of the curve: proximal thoracic rotation (apical) (PTR), main thoracic rotation (apical) (MTR), lumbar rotation (apical) (LR).

Finally, to determine the differences between the clusters in terms of clinically measurable variables, the clinical measurements of the patients at PO, EP, and 2Y were measured and compared between clusters at each time-point. These variables are the parameters that either can be directly measured on the spinal X-rays or via a commercial software (SterEOS 2D/3D, EOS imaging, Paris, France). The visual descriptions of each of these measurements are presented in Fig. 2. These clinical parameters are proximal thoracic Cobb (PTC), proximal thoracic rotation (apical) (PTR), main thoracic Cobb (MTC), main thoracic rotation (apical) (MTR), lumbar Cobb (LC), lumbar rotation (apical) (LR), thoracic kyphosis (TK both between T1-T12 and T4-T12), lumbar lordosis (LL), pelvic incidence (PI), sacral slope (SS), pelvic tilt (PT), frontal balance (FB), and sagittal balance (SB). Data normality was tested using the Shapiro-Wilk test and the sample size was determined for a test of comparison of the means between the clusters for a type I error of 0.05 and type II error of 90%.

## Results

**Figure 3:**
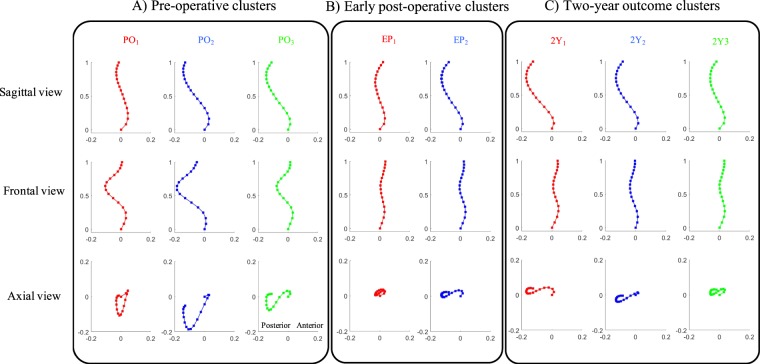
Groups identified using the K-means clustering analysis of the 3D spinal curvatures at three different time-points. The projections of the cluster centers of the spinal curves are shown in the sagittal, frontal, and axial planes for cluster 1 (red), cluster 2 (blue), and cluster 3 (green) at three different time-points (**A**) The pre-operative (PO), (**B**) early post-operative (EP), and (**C**) two-year outcome (2Y). The PO 3D curve patterns are characterized as follows. These characteristics were in the sagittal view cluster 1: hypokyphotic range with no proximal kyphosis, cluster 2: normal-hypokyphotic range with no proximal kyphosis and a high inflection point (T11), cluster 3: normal-hypokyphotic range with proximal kyphosis and a lower level inflection point (L1). In frontal view cluster 1: balanced, cluster 2: unbalanced, and cluster 3: balanced. In the axial view cluster 1: closed V shape, cluster 2: V shaped, and cluster 3: S shaped. The two EP clusters in the three views are shown in (**B**). The sagittal characteristics of cluster 1 are flat, and of cluster 2 are normal-hypo kyphotic range with proximal kyphosis and a low-level inflection point (L1). The Frontal characteristics are as follows. Cluster 1: lateral shift and cluster 2: balanced. The axial characteristics are cluster 1: closed loop, cluster 2: S shape. The sagittal characteristics are: cluster 1: hyperkyphosis backward shift, for cluster 2: normal-hypo kyphotic, and for cluster 3: flat. The frontal characteristics are cluster 1: unbalance, cluster 2: balance, and cluster 3: unbalance. The axial characteristics are cluster 1: S shaped, cluster 2: S shaped, cluster 3: S shaped-closed.

**Figure 4:**
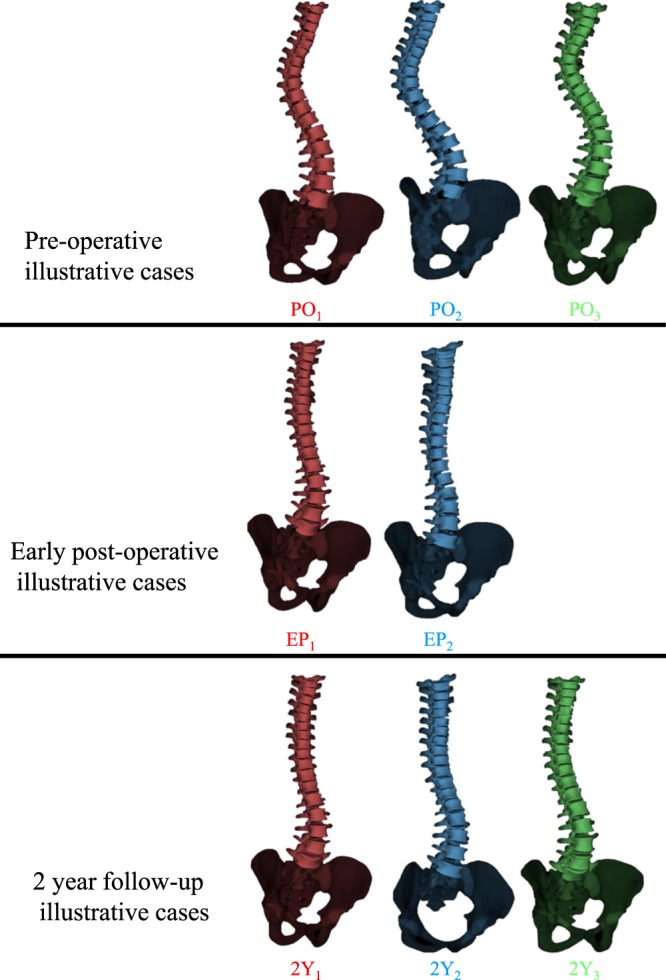
3D illustration of the clusters at pre-operative, early post-operative, and two-year follow-up.

Three clusters are identified at PO_i_, i = 1, 2, 3. The sagittal, frontal, and axial views of the spinal curvature in each cluster are shown in Fig. 3(A). The EP_j_ spines are grouped in two clusters, j = 1, 2, Fig. 3(B). The spines at 2Y_k_ follow-up are clustered into three groups, k = 1, 2, 3, shown in Fig. 3(C). Figure 4 shows an illustrative case in 3D for each of these curve types at different time-points.

Table 1 summarizes the average and standard deviation (SD) of the clinical parameters of the PO, EP, and 2Y clusters. The statistical methods and the significant levels are shown in Table 1. Significant differences were observed between PO clusters in PTR, MTR, and TK, between EP clusters in MTC, LC, TK, and SB, and between 2Y clusters in LC, LR, TK, and SB (Table 1).

**Table 1:** Clinical measurements of the clusters at pre-operative (PO), early post-operative (EP), and two-year follow-up (2Y).

PO	PTC	PTR	MTC	MTR	LC	LR	TK T1-T12	TK T4-T12	LL L1-S1	PI	SS^■^	PT^■^	FB	SB
1 n = 24	6.6 ± 16.0	0.8 ± 0.5	57.6 ± 7.5	−8.4 ± 6.6	37.6 ± 6.5	8.1 ± 5.2	19.6 ± 10.6^§^	7.6 ± 10.6^§^	52.3 ± 11.4	54.7 ± 13.1	47.1 ± 9.1	7.6 ± 7.8	0.8 ± 22.1	0 ± 16.4^§^
2 n = 21	10.4 ± 16.8	0.0 ± 0.7	60.3 ± 10.3	−14.0 ± 7.0	42.1 ± 9.1	11.7 ± 5.3	32.6 ± 11.6*	19.2 ± 11.6	59.9 ± 9.9	51.0 ± 14.4	45.8 ± 11.0	5.2 ± 8.5	4.3 ± 19.8^†^	−18 ± 18.4*
3 n = 19	16.5 ± 16.3	−4.0 ± 2.0*^§^	58.9 ± 7.6	−4.6 ± 5.7^§^	40.9 ± 6.7	6.4 ± 5.7	24.4 ± 11.5	18.5 ± 11.5	46.5 ± 9.2	45.0 ± 12.5	35.7 ± 9.0	9.3 ± 7.7	0.5 ± 20.2	−7 ± 18.8
*P* values	—	0.034	—	0.046	—	—	0.030	0.035	—	—	—	—	—	—
**EP**	**PTC** ^**■**^	**PTR** ^**■**^	**MTC**	**MTR**	**LC**	**LR** ^**■**^	**TK T1-T12**	**TK T4-T12**	**LL L1-S1**	**PI**	**SS** ^**■**^	**PT** ^**■**^	**FB**	**SB**
1 n = 41	1.6 ± 4.9	0 ± 0.1	13.5 ± 13.2^§^	2.3 ± 6.9	14.3 ± 14.8^§^	4.4 ± 5.6	27.1 ± 13.7	16.6 ± 9.3	45.3 ± 18.4	48.7 ± 20.3	37.5 ± 14.6	11.2 ± 8.4	6 ± 15.4	−8 ± 13.3^§^
2 n = 23	0 ± 1.8	0 ± 0.8	23.5 ± 14.3*	6.6 ± 7.0	23.2 ± 14.5*	9.0 ± 6.4	34.4 ± 13.0	26.0 ± 8.2*	54.8 ± 18.9	49.7 ± 17.2	40.1 ± 14.4	9.6 ± 7.2	2 ± 12.5	4 ± 14.0*
*P* values	—	—	0.038	—	0.040	—	—	0.022	—	—	—	—	—	0.019
**2Y**	**PTC** ^**■**^	**PTR** ^**■**^	**MTC**	**MTR** ^**■**^	**LC**	**LR** ^**■**^	**TK T1-T12**	**TK T4-T12**	**LL L1-S1**	**PI**	**SS** ^**■**^	**PT** ^**■**^	**FB**	**SB** ^**■**^
1 n = 24	1.6 ± 5.7	0.0 ± 1.3	18.4 ± 12.1	−1.1 ± 5.1	13.2 ± 11.3	0.6 ± 1.2	37.6 ± 10.1	23.3 ± 6.5	57.4 ± 9.5	49.3 ± 13.8	43.2 ± 8.5	6.1 ± 9.4	0 ± 14.5	−10 ± 10.4
2 n = 17	0.1 ± 6.8	0.1 ± 1.8	17.1 ± 11.9	−1.0 ± 4.1	18.5 ± 11.9	2.8 ± 1.8*	39.8 ± 8.5^†^	23.9 ± 5.6	50.2 ± 8.8	50.0 ± 12.0	44.8 ± 10.2	7.8 ± 10.1	8 ± 12.1	−14 ± 8.1
3 n = 23	4.3 ± 5.8	0.3 ± 0.0	18.1 ± 15.4	−1.5 ± 2.4	21.3 ± 14.4*	2.3 ± 1.3*	29.4 ± 9.5	27.3 ± 8.7	58.1 ± 8.6	48.2 ± 12.5	41.9 ± 11.3	5.3 ± 7.8	5 ± 16.6	1 ± 12.8^§^
*P* values	—	—	—	—	0.042	(1,2)0.038 (1,3)0.034	0.038	—	—	—	—	—	—	0.010

Type I error is set to 0.05. PTC: proximal thoracic Cobb, PTR: proximal thoracic rotation (apical), MTC: Main thoracic Cobb, MTR: Main thoracic rotation (apical), LC: lumbar Cobb, LR: lumbar rotation (apical), TK: thoracic kyphosis, LL: lumbar lordosis, PI: pelvic incidence, SS: sacral slope, PT: pelvic tilt, FB: frontal balance, SB: sagittal balance. The variables marked by ■ normally distributed and were tested by ANOVA –posthoc Tukey’s. The reset of variables were tested using Kruskal-Wallis- Dunn test. *Significantly different from cluster 1, ^§^Significantly different from cluster 2, ^†^Significantly different from cluster 3.

The fusion levels and their assigned group number are listed in Table 2. A total number of 8 distinct fusion levels are identified. Two patients were fused between T3-T11 vertebral levels and one between T2-T10 levels. Due to the small number of patients with these two fusion levels, these patients are removed from the analysis (total of 3 patients). The remaining 64 patients and 6 fusion groups are included in the following analysis.

**Table 2:** Indication of the upper and lower instrumented vertebrae (UIV, LIV) position.

Group	# patients	UIV	LIV
G1	8	T2	T12
G2	10	T3	T12
G3	14	T4	T12
G4	12	T2	L1
G5	8	T3	L1
G6	12	T4	L1
G7*	2	T3	T11
G8*	1	T2	T10

*Eliminated fusion levels due to small number of patients in these groups.

Classifying the 3D spinal curves into a limited number of PO and EP clusters, along with identifying a handful of distinct fusion levels provide the opportunity for identifying a limited number of *treatment paths*. Figure 1 shows the framework for determining these *treatment paths*. A patient example is shown in Figure 1; the patient belonged to cluster 3 at PO, was fused from T4 to L1 (Group 6, Table 2) and belonged to the cluster 1 at EP, thus was assigned to the *treatment path* PO_3_-FE_1_-F_6_. The two-year outcome cluster as determined by the 3D spinal curve belongs to cluster 2Y_1_. The number of patients in each 2Y outcome cluster for all the existing *treatment paths* are reported in Table 3.

**Table 3:** Identification of treatment outcome paths and the number of patients in the outcome clusters for the patients included in the study analysis.

Path	PO	EP	Fusion Group	2Y1	2Y2	2Y3
1	1	1	2,6	4	1	0
2	1	1	3,5	0	1	6
3	1	2	1	0	1	0
4	1	2	3,4	1	1	4
5	1	2	2	0	1	1
6	1	2	4,6	3	1	0
7	2	1	1,2	5	1	1
8	2	1	5,6	1	5	0
9	2	1	4	0	0	1
10	2	1	3	1	0	0
11	2	2	2	4	0	0
12	2	2	5	0	0	1
13	2	2	4	0	1	0
14	3	1	3	0	5	1
15	3	1	1	1	0	0
16	3	1	4	0	0	5
17	3	1	6	5	1	0

PO: pre-operative, EP: early post-operative, and 2Y: two-year outcome.

In order to address whether the *treatment paths* can significantly predict the 2Y clusters, a multinomial logistic regression was performed. The details of the multinomial logistic regression for association between the assigned *treatment path* (predictors) and the 2Y outcomes (predicted) are described in the Online Supplementary Information. Likelihood ratio tests of the regression model show significant association between the predicted and predictor variables, i.e., *treatment paths* and 2Y outcomes, χ^2^ = 132.52, p = 8.3825e-11.

**Figure 5:**
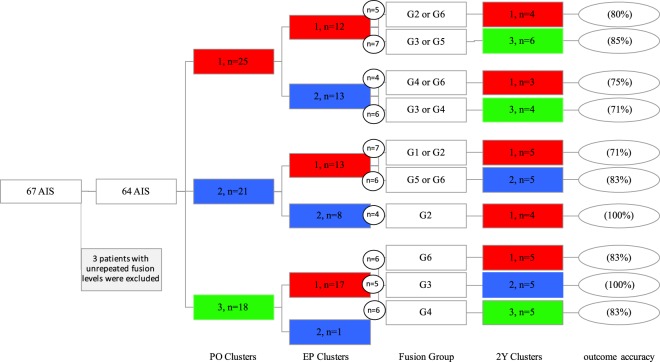
The number of patients in each of the clusters at different time-points and the treatment paths. The total number of patients who had the same treatment path, i.e., same PO cluster, EP cluster, and fusion group is shown in the small circles. The percentage of patients in each treatment path who are classified in the same 2Y outcome is shown in the ovals. Only the treatment paths with three or more patients are shown. The complete path- assignment for all the patients is presented in Table 3.

The number of patients who belonged to the same *treatment path* and 2Y cluster are calculated in Fig. 5. In instances where different fusion groups resulted in the same 2Y outcomes, the fusion groups are merged. Out of the total number of 17 existing *treatment paths*, a total number of 10 paths had more than two patients (Fig. 5). These 10 *treatment paths* include 56 patients. At least 70% of the patients who belonged to each of these 10 *treatment paths* had the same two-year outcome (Fig. 5). As an example, a total number of 6 patients belonged to the P_3_-FE_1_-F_6_ path (path 17), out of which 5 (83%) were clustered in 2Y_1_. The *treatment path* of these patients is also shown in Fig. 1.

## Discussion

Spinal fusion in AIS aims to realign the vertebrae in order to reduce the frontal deformity and curve rotation while imparting *adequate* sagittal profile^31^. New surgical maneuvers and instrumentation techniques allow for greater deformity correction of the curve in the three anatomical planes^32^. While much emphasis has been put on the pre-operative shape of the spine to guide the surgical decision-making, it has not been quantitatively determined whether a pathway which incorporates both the *objectively* identified pre-operative shape of the spine and the biomechanical changes in the spinal curve during the surgery can determine the long-term outcomes of the operation in AIS patients.

The 3D analysis of the spine has shown promising results in terms of determining subgroups of patients pre-operatively^33,34^. Given the importance of the 3D spinal curvature in postural assessment of the AIS patient^35,36^, a low-dimensional model that facilitates comparison between the 3D spinal shapes can improve the curve classification in AIS patients. The spinal evaluation in the three anatomical planes has underlined the parameters associated with the surgical outcome in AIS, which were not evident via the 2D evaluation of the spine^9,37^. Moreover, considering the interconnection of the pre- and post-operative 3D shape of the spine and the surgical outcomes, in the current study, we developed a quantitative pathway (patent pending) that can determine the outcome of the spinal surgery in AIS as a function of the 3D pre-operative spinal curve patterns as well as the modifiable factors during the surgery.

Here, to explore such pathways: (1) an objective classification of the 3D spinal curve was developed, and (2) the minimum key surgical parameters were identified. Regarding (1), the advantage of the clustering method based on the 3D shape of the isotropically scaled spines as opposed to defining the curves, based on a number of geometrical variables is reduced computational time and error. This allows recognizing the 3D patterns of the spinal curvature as opposed to the previous methods that extracted 3D variables of the curve for classification purposes^33^. Regarding (2), the 3D shape of the spine early after surgery in addition to the fusion level was used for characterizing the surgically induced changes in the spine. Using (1) and (2) in conjunction, a low-dimensional framework for assigning the most plausible outcome in terms of the 3D spinal curvature was introduced and evaluated in a group of 64 AIS patients with a thoracic curve. This approach determined the 3D shape of the spine in two-year follow-ups in 88% of the patients with an accuracy of at least 70%.

Selecting the fusion level is an ongoing debate in the surgical treatment of AIS^38–40^. The current clinical classification system and its modified versions attempt to address this issue^13,41,42^, yet complications and negative outcomes have challenged the surgical decision-making based on this guidelines^24,43,44^. Because the fusion level impacts the cost and time of the surgery^45,46^, patient’s balance and posture after operation^39,47^, spinal flexibility, and disc degeneration^41,48^ a quantifiable standardized tool for choosing the fusion levels is of critical need. Our proposed method lays out a pathway for outcome determination based on the shape of the spine before and after the surgery as the fusion levels changes (Fig. 1). As the clustering of the 2Y outcomes clearly distinguishes poor outcomes in term of the sagittal and frontal imbalances and residual curve at two-year post-operative, considering the *treatment paths* that results in a more favorable 2Y outcome can be achieved by selecting specific fusion levels (F). Comparing the clinical outcomes (2Y clusters) shows that achieving 2Y_2_ at two-year follow-up is advantageous over clusters 1 and 3 due to the smaller change in the proximal junctional kyphosis, smaller residual lumbar curve, and both frontal and sagittal balances closer to the normative values^49^ (Fig. 3), thus favoring the treatment paths ending to the 2Y_2_. As it is seen the fusion level can affect this outcome; for example, patients in clusters PO_3_ and EP_1_ can have 2Y_2_ if they were to have fusion levels G3 (T4-T12) (Fig. 5), while other fusion levels (G6 (T4-L1) and G4(T2-L1)) resulted in a different 2Y outcome.

The framework introduced here promises a surgical decision-making tool that can be personalized for physicians based on their preferred techniques. The rod length and curvature (using the EP clusters) can be determined prior to surgery to reduce the operation room time and number of staff which in turns reduces the surgical cost as well as blood loss and risk of infection^44,50,51^. Moreover, as the patients’ expectation plays an important role in their satisfaction, self-image, and quality of life after surgery^52–54^, by exploring the possible outcomes, a more comprehensive expectation of the surgical outcomes can be achieved by the patient, patient’s family, and their surgeon as they go through the process of surgery. This framework can be used as an assistive tool for the surgeons to modify several factors during the surgery in order to minimize the differences between the *optimal* and *possible* outcomes, similar to minimizing *regret* in decision theory^55,56^. Formulation of this decision-making problem with an embedded optimization approach will require additional factors that can possibly impact the outcomes.

The limitations of the current work include single-center and small patient dataset. This at some level limits incorporating various surgical technique, implant density, and variation in the implant including the material properties, rod diameter, and screw types. Additional surgical factors, which may vary between the surgeons and hospitals, remain to be evaluated via a larger multi-center study. While the impact of other surgical factors such as both magnitude and technique of the thoracic de-rotation, LIV alignment correction, number of osteotomies, and the reduction technique were not included, the early post-operative clustering of the spinal alignment in part accounts for these variables. A quantitative follow-up analysis of the spinal alignment as they relate to these surgical procedures is the subject of another study. Detailed analysis of the spinal curvature as considers the geometrical differences in the 3D spinal curve will be investigated in a larger database. Only patients with a right thoracic curve, the most common AIS deformity^49^, were included in this study. Application of a similar framework for surgical prediction outcomes in other types of spinal deformities and adult spinal surgical planning should be explored. As for any classification technique, the patients with *borderline* characteristics can adversely impact the outcome prediction. Yet, the K-means clustering classification in our cohort identified the outcomes with an accuracy of 70% or higher for 88% of the patients. More detailed clustering methods and its impact on the oucome prediction accuracy will be investigated. For a more heterogeneous cohort, Fuzzy clustering offers solution by assigning membership grades and further developing a probabilistic path for determining the outcome groups^57^. Despite the significant relationship between the intraoperative radiographs and the surgical outcome of AIS spinal fusion^58^, a study that associates the imparted intraoperative changes in the spine and the early post-operative alignment in a larger database can better guide surgical decision making.

The framework proposed here has an important implication in robotic surgery where a more *quantifiable* surgical goal can be established. Superimposition of both pre-operative and the aimed post-operative shape of the spine on the intraoperative images, not only can facilitate the screw placement but also guide imparting the required changes in the spinal profile in 3D. Furthermore, quantifying the trajectory of the changes in the spinal vertebrae position while quantifying the spinal flexibility through MRI^59^ can closely calculate the required forces during the correction surgery. Quantifying these components can lead to a promising future for robotic surgery in pediatric spinal surgery.

## Electronic supplementary material

Supplementary information

## References

[1] Parent S, Newton PO, Wenger DR (2005). Adolescent idiopathic scoliosis: etiology, anatomy, natural history, and bracing. Instr Course Lect.

[2] Burton MS (2013). Diagnosis and treatment of adolescent idiopathic scoliosis. Pediatr Ann.

[3] Cheng JC (2015). Adolescent idiopathic scoliosis. Nat Rev Dis Primers.

[4] Bridwell, K.H. Spinal instrumentation in the management of adolescent scoliosis. *Clin Orthop Relat Res*. 64–72 (1997). 9020207

[5] Schulz J (2014). Optimal radiographical criteria after selective thoracic fusion for patients with adolescent idiopathic scoliosis with a C lumbar modifier: does adherence to current guidelines predict success?. Spine (Phila Pa 1976).

[6] Giudici F (2017). Determinants of the biomechanical and radiological outcome of surgical correction of adolescent idiopathic scoliosis surgery: the role of rod properties and patient characteristics. Eur Spine J.

[7] Marks M (2012). Postoperative segmental motion of the unfused spine distal to the fusion in 100 patients with adolescent idiopathic scoliosis. Spine (Phila Pa 1976).

[8] Pasha, S., Flynn, J. M. & Sankar, W. N. Outcomes of selective thoracic fusion for Lenke 1 adolescent idiopathic scoliosis: predictors of success from the sagittal plane. *Eur Spine J* (2018). 10.1007/s00586-018-5553-9 29564609

[9] Pasha S (2017). Timing of Changes in Three-Dimensional Spinal Parameters After Selective Thoracic Fusion in Lenke 1 Adolescent Idiopathic Scoliosis: Two-Year Follow-up. Spine Deform.

[10] Ilharreborde B (2018). Sagittal balance and idiopathic scoliosis: does final sagittal alignment influence outcomes, degeneration rate or failure rate?. Eur Spine J.

[11] Enercan M (2016). Does It Make a Difference to Stop Fusion at L3 Versus L4 in Terms of Disc and Facet Joint Degeneration: An MRI Study With Minimum 5 Years Follow-up. Spine Deform.

[12] Lenke LG, Edwards CC, Bridwell KH (2003). The Lenke classification of adolescent idiopathic scoliosis: how it organizes curve patterns as a template to perform selective fusions of the spine. Spine (Phila Pa 1976).

[13] Lenke LG (2001). Adolescent idiopathic scoliosis: a new classification to determine extent of spinal arthrodesis. J Bone Joint Surg Am.

[14] King HA, Moe JH, Bradford DS, Winter RB (1983). The selection of fusion levels in thoracic idiopathic scoliosis. J Bone Joint Surg Am.

[15] Pasha S, Cahill PJ, Dormans JP, Flynn JM (2016). Characterizing the differences between the 2D and 3D measurements of spine in adolescent idiopathic scoliosis. Eur Spine J.

[16] Pasha, S., Ecker, M. & Deeney, V. Considerations in sagittal evaluation of the scoliotic spine. *Eur J Orthop Surg Traumatol* (2018). 10.1007/s00590-018-2175-1 29541842

[17] Hosseinpour-Feizi H, Soleimanpour J, Sales JG, Arzroumchilar A (2011). Lenke and King classification systems for adolescent idiopathic scoliosis: interobserver agreement and postoperative results. Int J Gen Med.

[18] Duong L (2009). Interobserver and intraobserver variability in the identification of the Lenke classification lumbar modifier in adolescent idiopathic scoliosis. J Spinal Disord Tech.

[19] Miyanji F, Pawelek JB, Van Valin SE, Upasani VV, Newton PO (2008). Is the lumbar modifier useful in surgical decision making?: defining two distinct Lenke 1A curve patterns. Spine (Phila Pa 1976).

[20] Niemeyer Thomas, Wolf Alexandra, Kluba Susanne, Halm Henry F., Dietz Klaus, Kluba Torsten (2006). Interobserver and Intraobserver Agreement of Lenke and King Classifications for Idiopathic Scoliosis and the Influence of Level of Professional Training. Spine.

[21] Chang KW (2011). Broader curve criteria for selective thoracic fusion. Spine (Phila Pa 1976).

[22] Cahill PJ (2014). Is there a role for the 5-degree rule in adolescent idiopathic scoliosis?. J Pediatr Orthop.

[23] Wang Y, Bunger CE, Wu C, Zhang Y, Hansen ES (2012). Postoperative trunk shift in Lenke 1C scoliosis: what causes it? How can it be prevented?. Spine (Phila Pa 1976).

[24] Ahmed SI, Bastrom TP, Yaszay B, Newton PO, Harms Study G (2017). 5-Year Reoperation Risk and Causes for Revision After Idiopathic Scoliosis Surgery. Spine (Phila Pa 1976).

[25] Ilharreborde B (2011). Angle measurement reproducibility using EOS three-dimensional reconstructions in adolescent idiopathic scoliosis treated by posterior instrumentation. Spine (Phila Pa 1976).

[26] Humbert L, De Guise JA, Aubert B, Godbout B, Skalli W (2009). 3D reconstruction of the spine from biplanar X-rays using parametric models based on transversal and longitudinal inferences. Med Eng Phys.

[27] Pasha, S. *et al*. Application of Low-dose Stereoradiography in *In Vivo* Vertebral Morphologic Measurements: Comparison with Computed Tomography. *J Pediatr Orthop*. 10.1097/BPO.0000000000001043 31503238

[28] Lloyd SP (1982). Least squares quantization in PCM. IEEE Transactions on Information Theory.

[29] Rousseeuw PJ (1987). Silhouettes: A graphical aid to the interpretation and validation of cluster analysis. Journal of Computational and Applied Mathematics.

[30] Croissant Y (2018). mlogit: Multinomial Logit Models. R package version.

[31] Pasha, S., Illhaborde, B. & Baldwin, K. Sagittal Spinopelvic Alignment after Posterior Spinal Fusion in Adolescent Idiopathic Scoliosis: a Systematic Review and Meta-Analysis. *Spine* (*Phila Pa 1976*) (2018). 10.1097/BRS.0000000000002736 29889799

[32] Yilmaz G (2012). Comparative analysis of hook, hybrid, and pedicle screw instrumentation in the posterior treatment of adolescent idiopathic scoliosis. J Pediatr Orthop.

[33] Sangole AP (2009). Three-dimensional classification of thoracic scoliotic curves. Spine (Phila Pa 1976).

[34] Duong L, Cheriet F, Labelle H (2006). Three-dimensional classification of spinal deformities using fuzzy clustering. Spine (Phila Pa 1976).

[35] Pasha S, Baldwin K (2018). Are we simplifying balance evaluation in adolescent idiopathic scoliosis?. Clin Biomech (Bristol, Avon).

[36] Pasha S (2014). Three-dimensional spinopelvic relative alignment in adolescent idiopathic scoliosis. Spine (Phila Pa 1976).

[37] Pasha S (2018). Relationships Between the Axial Derotation of the Lower Instrumented Vertebra and Uninstrumented Lumbar Curve Correction: Radiographic Outcome in Lenke 1 Adolescent Idiopathic Scoliosis With a Minimum 2-Year Follow-up. J Pediatr Orthop.

[38] Fischer CR (2018). Optimal Lowest Instrumented Vertebra for Thoracic Adolescent Idiopathic Scoliosis. Spine Deform.

[39] Zhao J (2018). Does spinal fusion to T2, T3, or T4 affects sagittal alignment of the cervical spine in Lenke 1 AIS patients: A retrospective study. Medicine (Baltimore).

[40] Rizkallah, M., Sebaaly, A., Kharrat, K. & Kreichati, G. Selecting the lowest instrumented vertebra in adolescent idiopathic scoliosis: Comparison of the Lenke, Suk, and Dubousset criteria. *Orthop Traumatol Surg Res* (2017). 10.1016/j.otsr.2017.12.007 29292125

[41] Bai J (2018). Selecting the LSTV as the Lower Instrumented Vertebra in the Treatment of Lenke Types 1A and 2A Adolescent Idiopathic Scoliosis: A Minimal 3-year Follow-up. Spine (Phila Pa 1976).

[42] Qin X (2016). Selecting the Last “Substantially” Touching Vertebra as Lowest Instrumented Vertebra in Lenke Type 1A Curve: Radiographic Outcomes With a Minimum of 2-year Follow-Up. Spine (Phila Pa 1976).

[43] Ramo BA, Richards BS (2012). Repeat surgical interventions following “definitive” instrumentation and fusion for idiopathic scoliosis: five-year update on a previously published cohort. Spine (Phila Pa 1976).

[44] Carreon LY (2007). Non-neurologic complications following surgery for adolescent idiopathic scoliosis. J Bone Joint Surg Am.

[45] Martin CT (2014). Increasing hospital charges for adolescent idiopathic scoliosis in the United States. Spine (Phila Pa 1976).

[46] Vigneswaran HT, Grabel ZJ, Eberson CP, Palumbo MA, Daniels AH (2015). Surgical treatment of adolescent idiopathic scoliosis in the United States from 1997 to 2012: an analysis of 20,346 patients. J Neurosurg Pediatr.

[47] Cho KJ, Lenke LG, Bridwell KH, Kamiya M, Sides B (2009). Selection of the optimal distal fusion level in posterior instrumentation and fusion for thoracic hyperkyphosis: the sagittal stable vertebra concept. Spine (Phila Pa 1976).

[48] Clement JL (2017). Five-year outcomes of the First Distal Uninstrumented Vertebra after posterior fusion for Adolescent Idiopathic Scoliosis Lenke 1 or 2. Orthop Traumatol Surg Res.

[49] Pasha, S. & Baldwin, K. Preoperative Sagittal Spinal Profile of Adolescent Idiopathic Scoliosis Lenke Types and Non-Scoliotic Adolescents: a Systematic Review and Meta-Analysis. *Spine* (*Phila Pa 1976*) (2018). 10.1097/BRS.0000000000002748 29927859

[50] Marks MC (2013). Surgical Site Infection in Adolescent Idiopathic Scoliosis Surgery. Spine Deform.

[51] Chan CY, Kwan MK (2016). Perioperative Outcome in Posterior Spinal Fusion for Adolescent Idiopathic Scoliosis: A Prospective Study Comparing Single Versus Two Attending Surgeons Strategy. Spine (Phila Pa 1976).

[52] Ding R, Liang J, Qiu G, Shen J, Li Z (2014). Evaluation of quality of life in adolescent idiopathic scoliosis with different distal fusion level: a comparison of L3 versus L4. J Spinal Disord Tech.

[53] Ersberg A, Gerdhem P (2013). Pre- and postoperative quality of life in patients treated for scoliosis. Acta Orthop.

[54] Hisam MA (2015). Does the Quality of Life in Operated Patients with Adolescent Idiopathic Scoliosis correspond with the RadiographicParameters?. Malays Orthop J.

[55] Bell DE (1982). Regret in decision making under uncertainty. Operations research.

[56] Loomes G, Sugden R (1982). Regret theory: An alternative theory of rational choice under uncertainty. Economic Journal.

[57] Dunn JC (1973). A Fuzzy Relative of the ISODATA Process and Its Use in Detecting Compact Well-Separated Clusters. Journal of Cybernetics..

[58] Lehman RA (2010). Do intraoperative radiographs in scoliosis surgery reflect radiographic result?. Clin Orthop Relat Res.

[59] Zanjani-Pour S, Winlove CP, Smith CW, Meakin JR (2016). Image driven subject-specific finite element models of spinal biomechanics. J Biomech.

