# Supplementary material for: Data-driven Classification of the 3D Spinal Curve in Adolescent Idiopathic Scoliosis with an Applications in Surgical Outcome Prediction
Source: Sci Rep. 2018 Nov 2;8:16296. doi: 10.1038/s41598-018-34261-6 (PMC6214965; doi:10.1038/s41598-018-34261-6)
Supplement: Supplementary file 1 — Supplementary information [file 41598_2018_34261_MOESM1_ESM.docx]

Title: Data-driven Classification of the 3D Spinal Curve in Adolescent Idiopathic Scoliosis with an Applications in Surgical Outcome Prediction

Authors: Saba Pasha^1,2^, John Flynn^1,2^

Affiliations:

^1^Division of Orthopedic Surgery, Children’s Hospital of Philadelphia, Philadelphia PA, 19141 8 USA ^2^Department of Surgery, University of Pennsylvania, Philadelphia PA, 19141 USA

Correspondence to: pashas@email.chop.edu

**Multinomial logistic regression model**

**Methods:** A multinomial logistic regression was used to determine the probability of the membership in the 2 year outcome clusters based on an independent categorical variable i.e. the treatment path (equation 1). A multimodal logistic regression, as opposed to the binary logistic regression, was used since the 2year outcomes were determined to have 3 clusters (Figure 1). The sample size requirement was verified based on Schwab, 2002.^1^ The (mlogit) package^2^ in R Studio (RStudio, Inc., Boston, MA) was used to fit the multimodal model.^3^

**Results:** The model fit was determined from the likelihood ratio test χ2= 132.52, p= 8.3825e-11. The McFadden Pseudo R2 showed a large effect size (R_McFadden^2= 0.94). The negative Log-Likelihood was -3.8883.

The logistic coefficients and standard errors are calculated. The outcome cluster 1 was specified as the baseline and the odds of membership in cluster 2 or 3 compared to cluster 1 was calculated.

|  | Coefficients | | Std. Errors | |
| --- | --- | --- | --- | --- |
|  | Intercept | Path | Intercept | Path |
| Cluster 2 (2Y) | -1.461 | 0.146 | 0.625 | 0.079 |
| Cluster 3 (2Y) | -3.540 | 0.350 | 0.865 | 0.087 |

- A one unit increase in the variable path is associated with the increase in the log odds of being in Cluster 2 vs. Cluster 1 in the amount of 0.147.
- A one unit increase in the variable path is associated with the increase in the log odds of being in Cluster 3 vs. Cluster 1 in the amount of 0.350.

The exponentiated coefficients defined as the ratio of the probability of choosing one 2Y Cluster over the probability of choosing the baseline Cluster (here Cluster 1) in the multinomial model was calculated as follows:

- The relative risk ratio for a one unit increase in the variable path is 1.158 for being in Cluster 2 vs. Cluster 1.
- The relative risk ratio for a one unit increase in the variable path is 1.419 for being in Cluster 3 vs. Cluster 1.

**References:**

1- Schwab, J. A. (2002). Multinomial logistic regression: Basic relationships and complete

problems. http://www.utexas.edu/courses/schwab/sw388r7/SolvingProblems/

2- Croissant, Y. (2011). Package ‘mlogit’. http://cran.r -project.org/web/packages/mlogit/index.html

3- RStudio Team (2016). RStudio: Integrated Development for R. RStudio, Inc., Boston, MA URL http://www.rstudio.com/.
